# Lateralization Effects on Cerebral Blood Flow in Patients With Unilateral Pulsatile Tinnitus Measured With Arterial Spin Labeling

**DOI:** 10.3389/fnhum.2020.591260

**Published:** 2020-11-17

**Authors:** Xiaoshuai Li, Pengfei Zhao, Xiaoyu Qiu, Heyu Ding, Han Lv, Zhenghan Yang, Shusheng Gong, Zhenchang Wang

**Affiliations:** ^1^Department of Radiology, Beijing Friendship Hospital, Capital Medical University, Beijing, China; ^2^Department of Otolaryngology Head and Neck Surgery, Beijing Friendship Hospital, Capital Medical University, Beijing, China

**Keywords:** auditory cortex, arterial spin labeling, pulsatile tinnitus, cerebral blood flow, lateralization effects

## Abstract

**Purpose:** To investigate cerebral blood flow (CBF) differences in patients with left- and right-sided pulsatile tinnitus (LPT and RPT) and healthy controls (HCs) to further explore the lateralization effects of PT using arterial spin labeling (ASL).

**Methods:** ASL data from 21 RPT patients, 17 LPT patients and 21 HCs were reviewed. Voxel-wise analysis and region of interest analysis were performed to explore differences in CBF among the three groups. Tinnitus Handicap Inventory (THI) score and tinnitus duration were obtained from each patient.

**Results:** Voxel-wise analysis showed that the CBF of the left inferior parietal gyrus was increased in both RPT and LPT patients compared with HCs (*P* < 0.001). Region of interest analysis revealed that the CBF of the left primary auditory cortex (PAC) was higher than that of the right, while the CBF of the right secondary auditory cortex (SAC) and auditory association cortex was higher than that of the left. These lateralization effects were present in all three groups. Compared with HCs, RPT patients showed increased CBF in the left PAC and SAC (PAC: *P* = 0.036; SAC: *P* = 0.012). No significant correlations were found between PT duration or THI score and altered CBF in above regions.

**Conclusion:** Increased CBF in the left inferior parietal gyrus is a common feature in both RPT and LPT patients, regardless of the perceived side of PT. The lateralization effects of auditory cortices may be a physiological characteristic of the normal brain. These findings may provide a new perspective for understanding the neurological pathophysiology of PT.

## Introduction

Pulsatile tinnitus (PT) is an abnormal auditory perception of internal blood flow sound, and its rhythm is consistent with the heartbeat (Holgate et al., 1977; Haraldsson et al., 2019). This condition accounts for approximately 4% of tinnitus patients (Otto et al., 2007). PT severely affects the patient’s physical health and quality of life, increasing the risk of anxiety, irritability, sleep disturbances, depression, and even suicide.

The etiologies of PT are relatively clear, and sigmoid sinus wall dehiscence with or without diverticulum is the most common and potentially curable abnormality associated with PT (Dong et al., 2015; Zhao et al., 2016). The sound is thought to originate from abnormal hemodynamics in the transverse and sigmoid sinuses and is transmitted to the inner ear through the dehiscent sinus wall. After transtemporal sigmoid sinus wall reconstruction, the sound can completely disappear (Eisenman, 2011; Zhang et al., 2019). Resting-state functional magnetic resonance imaging (fMRI) studies on PT have found abnormal neuronal activity in multiple regions (Han et al., 2014, 2015; Lv et al., 2016b) and increased functional connectivity between the auditory and non-auditory brain networks (Lv et al., 2016a, 2017). These findings support the presence of pathophysiological changes in the brains of PT patients.

Currently, the lateralization effect of PT has remained unclear. Both animal and human studies have indicated that auditory input can lead to increased spontaneous neuronal activity (Kaltenbach et al., 2004; Barascud et al., 2016). Some fMRI studies showed that the activation of the auditory cortices was lateralized in patients with left- and right-sided non-PT (Smits et al., 2007; Lanting et al., 2008). Most previous studies on PT included only right-sided PT (RPT) patients (Han et al., 2014, 2015; Lv et al., 2016a,b, 2017). The only study on the lateralization effects of PT found difference in functional connectivity characteristics within auditory network, and between auditory network and other brain networks, including self-perceptual network, executive control network and limbic network in left-sided PT (LPT) and RPT patients (Lv et al., 2018). Therefore, the lateralization effects of PT may be related to different neuronal activity in PT patients.

Since neuronal activity is closely related to brain perfusion (Gusnard et al., 2001; Raichle and Mintun, 2006; Lanting et al., 2009), in the case of PT, this activity may correspond to altered cerebral blood flow (CBF). Arterial spin labeling (ASL) is currently the only non-invasive perfusion imaging technique that uses magnetically labeled arterial blood water as an endogenous tracer (Detre et al., 1992). Blood flow is an important element of neuronal physiological activity (Gorelick et al., 2011). ASL can non-invasively measure CBF, which is coupled with regional neuronal activity (Lanting et al., 2009). Compared with the blood oxygenation level dependent (BOLD) signal, which is affected by draining veins, ASL provides higher spatial specificity to neuronal activity due to the capillary origin of ASL signal (Chen et al., 2015). Moreover, unlike BOLD, CBF is linearly related to brain oxidative metabolism (Chen et al., 2015). These factors indicate that ASL can be used to assess potential neuronal activity. Traditional perfusion imaging techniques, such as CT perfusion (CTP) imaging, dynamic susceptibility contrast (DSC), and dynamic contrast-enhanced (DCE) MRI, are always invasive. In contrast, ASL has the advantages of non-invasiveness, immediate repeatability, and simplicity and is increasingly used in functional imaging studies.

This study aimed to detect CBF differences in patients with LPT and RPT and healthy controls (HCs) to further explore the lateralization effects of PT using the technique of three-dimensional pseudo-continuous ASL (3D-pcASL). Thus, an exploratory voxel-wise analysis of CBF changes was performed among the three groups. Furthermore, we specifically investigated the lateralization effects of bilateral auditory cortices by region of interest (ROI) analysis.

## Materials and Methods

### Participants

A prospective study was conducted on PT patients hospitalized in the otolaryngology head and neck surgery department of our institution between September 2017 and January 2020. All patients showed unilateral pulse-synchronous sound, which disappeared after compressing the ipsilateral jugular vein. The audiology, laboratory, tympanic membrane, otoscope, and digital subtraction angiography (DSA) examination results were normal in each patient. Sigmoid sinus wall dehiscence with or without diverticulum was confirmed as the main etiology by CT arteriography/venography, and the sound was significantly reduced or disappeared after surgical reconstruction (Eisenman, 2011; Wang et al., 2014). ASL acquisition was performed before the surgery. Finally, this study enrolled 38 patients with unilateral PT, including 21 RPT patients and 17 LPT patients. The Tinnitus Handicap Inventory (THI) score obtained from each patient was used to evaluate the severity of PT.

Twenty-one gender-, age-, handedness-, and education level-matched volunteers were included in HC group through local community advertising. Participants were excluded if they suffered from non-PT, hearing loss (hearing thresholds more than 25 dB at any six different frequencies between 0.25 and 8 kHz), hyperacusis, stroke, head injury, tumor, other psychiatric and neurological disorders, systemic diseases (hypertension, hyperlipidemia, diabetes), drug and alcohol abuse in the last three months or MRI contraindications. The study protocol was approved by the Medical Research Ethics Committee in our institution. Each participant signed an informed consent form before MRI scanning.

### MRI Data Acquisition

All participants were scanned by a GE Discovery MR750W 3.0T MRI scanner with a standard head coil. High-resolution structural T1-weighted images were acquired as follows: inversion time (TI) = 450 ms; repetition time (TR) = 8.5 ms; echo time (TE) = 3.3 ms; slices = 156; slice thickness = 1 mm without gap; field of view (FOV) = 240 mm × 240 mm; acquisition matrix = 256 × 256; flip angle (FA) = 12°.

The ASL perfusion images were obtained using a 3D-pcASL sequence with fast spin-echo acquisition and background suppression as follows: TR/TE = 4854/10.7 ms; FOV = 240 mm × 240 mm; slices = 36; slice thickness = 4 mm without gap; FA = 111°; post-label delay (PLD) = 2025 ms; number of excitations = 3; in-plane resolution = 3.37 mm × 3.37 mm. Earplugs were used to reduce imaging noise for each participant. Each participant was required to close their eyes, stay awake, lie quietly and think of nothing during ASL data acquisition. To exclude images with artifacts in subsequent analysis, all image data were visually inspected.

### Data Preprocessing

The Statistical Parametric Mapping (SPM) (version 12)^1^ software package in MATLAB R2013b (MathWorks, Natick, MA, United States) was used to preprocess the data. The CBF maps were calculated from the ASL difference images and the reference images using a single-compartment model. The calculation process of CBF has been specifically described in a previous study (Wang et al., 2020). First, the one-step registration method was used to spatially normalize the CBF map of each participant to a PET template in the Montreal Neurological Institute (MNI) space. The voxel size of resampling was 2 mm × 2 mm × 2 mm. Then, the standardization of CBF was normalized by dividing the mean CBF of the whole brain to find smaller CBF difference (Aslan and Lu, 2010). Finally, the standardized CBF map was smoothed using a Gaussian kernel (8 mm full width at half maximum). The smoothed CBF image was directly used for whole brain voxel-wise analysis in SPM12.

The auditory cortex is involved in auditory information processing (Giroud et al., 2020). Several fMRI studies have confirmed changed neuronal activity of the auditory cortex in tinnitus patients (Smits et al., 2007; Lanting et al., 2014). A PET study showed that higher activity of the left primary auditory cortex (PAC) was a robust feature in tinnitus patients (Langguth et al., 2006). Geven et al. further found that in chronic tinnitus patients, the activity of the left PAC was higher than that of the right, while the activity of the right auditory association cortex (AAC) and secondary auditory cortex (SAC) was higher than that of the left (Geven et al., 2014). Based on these findings, we conducted an ROI analysis on six auditory cortical areas defined by Brodmann Areas (BAs) template: the bilateral BA41, BA42, and BA22, which represent the bilateral PAC, SAC, and AAC, respectively. The CBF value was extracted from each auditory cortex in the three groups. The lateralization index (LI) was used to evaluate the lateralization effect of auditory cortices in each group, which was defined as: (left – right)/(left + right) (Seghier, 2008). The LI ranged from −1 to +1. A positive LI indicated that the auditory cortex was lateralized to the left, and a negative LI indicated that the auditory cortex was lateralized to the right.

### Statistical Analysis

#### Demographic and Clinical Data Analysis

Differences in demographic data among the three groups (RPT, LPT, and HC) were analyzed using one-way analysis of variance (ANOVA) and Fisher’s exact test in SPSS 22.0 software (Chicago, IL, United States). Differences in clinical data between the PT groups were analyzed using two-sample *t*-test. P < 0.05 was considered statistically significant.

#### Voxel-Wise Analysis

To determine the CBF difference among the three groups, we performed one-way analysis of covariance (ANCOVA) with gender and age as covariates (voxel-wise uncorrected *P* < 0.001). A cluster-level familywise error (FWE) correction with *P* < 0.05 was performed for multiple comparisons, with a minimum cluster size of 317 voxels. The CBF value of the three groups was extracted for *post hoc* intergroup comparisons and corrected with the Bonferroni method in SPSS 22.0 software. The significance threshold was set for *P* < 0.05.

#### ROI Analysis

The ROI analysis was performed in SPSS 22.0 software. A paired-sample *t*-test was used to compare CBF differences in the bilateral PAC, SAC, and AAC in each group. One-way ANOVA was used to compare the CBF difference of each auditory cortex among the three groups, followed by *post hoc* intergroup comparisons (Bonferroni correction). The LI differences in PAC, SAC, and AAC among the three groups were conducted by one-way ANOVA. One-sample *t*-test with 0 as the comparison point was used to explore the significance of the LI of each auditory cortex in the three groups. *P* < 0.05 was set as the significance threshold.

Pearson correlation analysis was used to explore potential correlations between the CBF and clinical data (THI score and PT duration) in PT patients.

## Results

### Demographic and Clinical Data

In this study, fifty-nine participants were included, including 21 RPT patients, 17 LPT patients, and 21 HCs. The age, gender, and education level of each group were matched (Table 1). All participants were right-handed. No significant differences in PT duration and THI score were found between RPT and LPT patients.

**TABLE 1:** Demographic and clinical data.

	RPT (*n* = 21)	LPT (*n* = 17)	HC (*n* = 21)	*P*-value
Age (years)	39.3 ± 10.2	37.5 ± 10.0	39.3 ± 9.7	0.826^a^
Gender (male/female)	2/19	3/14	2/19	0.689^b^
Tinnitus side (right/left)	21/0	0/17	NA	NA
Education (years)	11.3 ± 3.7	10.8 ± 2.6	12.6 ± 3.6	0.264^a^
Handedness	21 right-handed	17 right-handed	21 right-handed	1.000^b^
PT duration (months)	36.5 ± 33.2	35.1 ± 26.6	NA	0.887^c^
THI score	62.7 ± 23.7	56.5 ± 18.1	NA	0.381^c^

*Data are expressed as the mean ± standard deviation. RPT: right-sided pulsatile tinnitus; LPT: left-sided pulsatile tinnitus; HC: healthy control; THI: Tinnitus Handicap Inventory; NA: not applicable.*

*^a^One-way ANOVA; ^b^Fisher’s exact test; ^c^Two-sample two-tailed t-test.*

### Voxel-Wise Analysis

**FIGURE 1:**
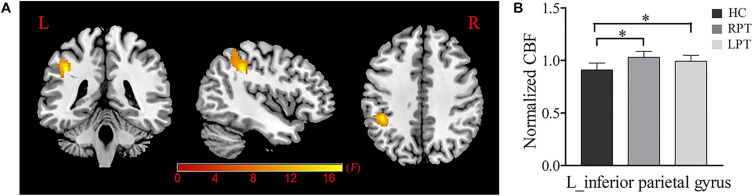
Normalized CBF difference among the RPT, LPT and HC groups. **(A)** One-way analysis of covariance (ANCOVA) showed significant group differences of the normalized CBF in the left inferior parietal gyrus (*P* < 0.05, FWE corrected). **(B)** *Post hoc* analysis indicated that the normalized CBF of the left inferior parietal gyrus was significantly increased in RPT and LPT patients compared with HCs. ^∗^Statistical significance with *P* < 0.05; RPT: right-sided pulsatile tinnitus; LPT, left-sided pulsatile tinnitus; HC, healthy control; CBF, cerebral blood flow; L, left.

A voxel-wise ANCOVA analysis showed that a significant CBF difference among the three groups was located at the left inferior parietal gyrus (IPG) (voxel-wise uncorrected *P* < 0.001, cluster-level *P* < 0.05 FWE corrected) (Figure 1). *Post hoc* analysis revealed that compared with HCs, increased CBF in the left IPG was found in RPT and LPT patients (*P* < 0.001). However, there were no significant CBF differences between RPT and LPT patients in this region.

### ROI Analysis

**FIGURE 2:**
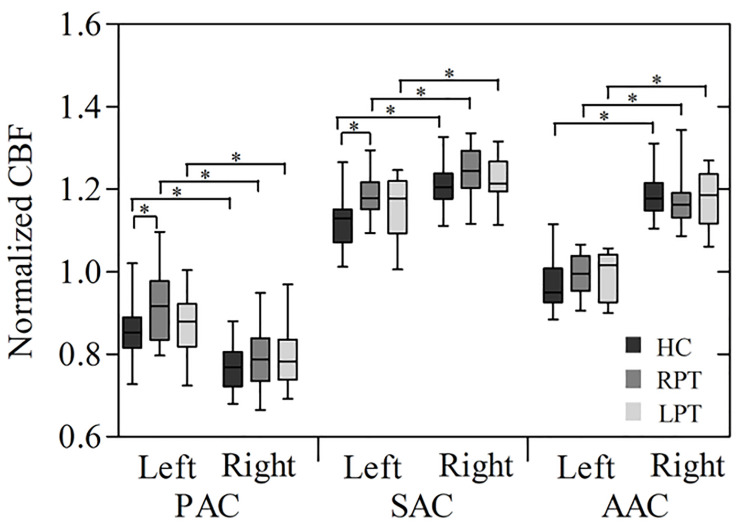
Box plot of normalized CBF measured with ROI analysis in the bilateral auditory cortices among the three groups. ^∗^Statistical significance with *P* < 0.05; PAC, primary auditory cortex; SAC, secondary auditory cortex; AAC, auditory association cortex; RPT, right-sided pulsatile tinnitus; LPT, left-sided pulsatile tinnitus; HC, healthy control; CBF, cerebral blood flow; ROI, region of interest.

The box plot in Figure 2 reveals the CBF of the bilateral auditory cortices in each group. In the three groups, the CBF of the left PAC was significantly higher than that of the right (HC: *P* < 0.001; RPT: *P* < 0.001; LPT: *P* = 0.001). In contrast, the CBF of the right SAC and AAC of the three groups was significantly higher than that of the left (SAC: HC, *P* < 0.001; RPT, *P* < 0.001; LPT, *P* = 0.002. AAC: HC, *P* < 0.001; RPT, *P* < 0.001; LPT, *P* < 0.001). One-way ANOVA revealed that significant CBF differences among the three groups were in the left PAC and SAC (PAC: *F* = 3.729, *P* = 0.030; SAC: *F* = 4.542, *P* = 0.015). *Post hoc* analysis revealed that RPT patients had increased CBF in the left PAC and SAC compared with HCs (Bonferroni corrected, PAC: *P* = 0.036; SAC: *P* = 0.012).

**FIGURE 3:**
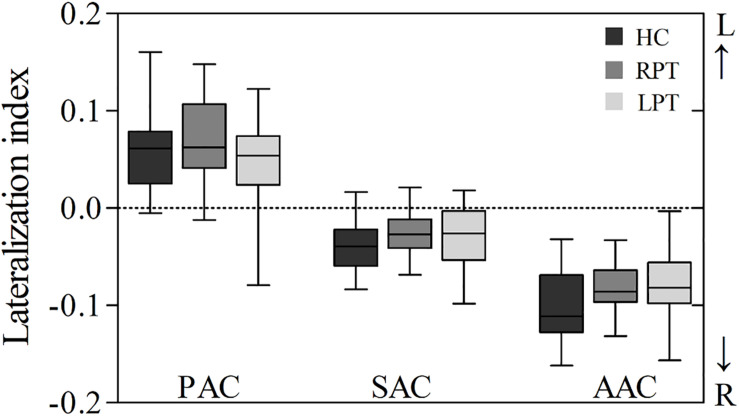
Box plot of LI in the auditory cortices of the three groups. A positive LI indicates that the auditory cortex was lateralized to the left, and a negative LI indicates that the auditory cortex was lateralized to the right. LI, lateralization index; PAC, primary auditory cortex; SAC, secondary auditory cortex; AAC, auditory association cortex; RPT, right-sided pulsatile tinnitus; LPT, left-sided pulsatile tinnitus; HC, healthy control.

The LI of PAC, SAC, and AAC in the three groups are showed in Figure 3 and Table 2. The PAC was lateralized to the left, while the SAC and AAC were lateralized to the right, which held for all three groups. Furthermore, the AAC exhibited stronger right lateralization than the SAC. No significant LI difference was found in PAC, SAC, and AAC among the three groups.

**TABLE 2:** The significance of the LI of the auditory cortices in the three groups.

	LI	*P*^a^
**PAC**		
HCs	0.054 ± 0.040	<0.001
RPT	0.070 ± 0.042	<0.001
LPT	0.046 ± 0.046	0.001
**SAC**		
HCs	−0.038 ± 0.029	<0.001
RPT	−0.025 ± 0.024	<0.001
LPT	−0.028 ± 0.033	0.002
**AAC**		
HCs	−0.099 ± 0.036	<0.001
RPT	−0.082 ± 0.022	<0.001
LPT	−0.081 ± 0.037	<0.001

*Data are expressed as the mean ± standard deviation. LI, lateralization index; PAC, primary auditory cortex; SAC, secondary auditory cortex; AAC, auditory association cortex; RPT, right-sided pulsatile tinnitus; LPT, left-sided pulsatile tinnitus; HC, healthy control.*

*^a^One-sample *t*-test.*

### Correlation Analysis

We did not find any statistical correlations between the PT duration or THI score and altered CBF in RPT and LPT patients.

## Discussion

To the best of our knowledge, the ASL technique was used for the first time to investigate brain perfusion differences in PT (LPT and RPT) patients and HCs to further explore the lateralization effects of PT. In this study, we demonstrated altered CBF in both RPT and LPT patients as well as lateralization effects of auditory cortices in the three groups, which provides a new perspective for understanding the neurological pathophysiology of PT.

In this study, compared with HCs, RPT and LPT patients exhibited increased CBF in the left IPG. IPG is associated with auditory perception and auditory memory (Gaab et al., 2003; Koelsch et al., 2009; Brancucci et al., 2011; Langsjo et al., 2012). A meta-analysis study showed that tinnitus is related to increased brain activity in this region (Cheng et al., 2020). De Ridder et al. (2014) further proposed that the IPG is a key component of the tinnitus network, which consists of IPG, parahippocampus, ventrolateral prefrontal cortex and auditory cortices. Since IPG is related to auditory perception and auditory memory in this network, it may be responsible for attributing the perception of the sound to an external source in tinnitus patients. Moreover, this region can modulate the activity of the auditory cortex (Paraskevopoulos et al., 2019) and participate in the auditory information processing (Bedny et al., 2010; Fiehler and Rösler, 2010). Transcranial magnetic stimulation of the IPG can transiently improve tinnitus (Vanneste et al., 2012), suggesting that this region is causally related to tinnitus perception. These findings indicate that IPG plays a key role in the neuropathology of tinnitus. An fMRI study found that compared with HCs, RPT patients showed increased neuronal activity in the IPG (Han et al., 2015). The increased neuronal activity can lead to increased brain perfusion (Lanting et al., 2009), which is in line with our finding. Furthermore, the IPG was involved in the abnormal functional connectivity in RPT patients (Lv et al., 2017). However, the above two studies on PT only included RPT patients. In this study, we included RPT and LPT patients, and found that compared with HCs, both PT subgroups (RPT and LPT) showed increased CBF in the left IPG. This indicated that increased CBF in the left IPG was a common feature for both RPT and LPT patients, regardless of the perceived side of PT.

The auditory cortex plays a key role in auditory information processing (Billig et al., 2019; Giroud et al., 2020). Previous studies confirmed the lateralization of the auditory cortex in structure and function (Hutsler and Galuske, 2003; Tervaniemi and Hugdahl, 2003; Dorsaint-Pierre et al., 2006). An fMRI study showed that the lateralization effects of the PAC and AAC existed not only in the tinnitus group, but also in the control group (Lanting et al., 2014). In a PET study, Geven et al. (2014) found that the metabolic activity of the left PAC was higher than that of the right PAC, while the metabolic activity of the right SAC and AAC was higher than that of the left SAC and AAC, which held true in both the bilateral tinnitus and control groups. This finding may indicate that the lateralization effects of the auditory cortices are a physiological characteristic of the normal brain. However, all the experimental groups in the above studies included non-PT patients, and it remains unclear whether the perceived side of tinnitus would affect the lateralization effects of the auditory cortex.

In this study, we included the RPT, LPT, and HC groups and found that the CBF of the left PAC was significantly higher than that of the right, while the CBF of the right SAC and AAC was significantly higher than that of the left. Compared with the study of Geven et al. (2014), we found significant differences in the CBF of the bilateral auditory cortices. This result may further demonstrate the existence of lateralization effects in the auditory cortices. Thus, we conclude that the lateralization effects of the auditory cortices in both the PT (RPT and LPT) and HC groups may be a physiological characteristic, which is independent of the perceived side of tinnitus and the presence of tinnitus.

In this study, in addition to the right AAC, the CBF of other auditory cortices in RPT and LPT patients was increased compared with HCs, and the CBF of the left PAC and SAC in RPT patients reached a significant difference. Normally, stimulating one side of the ear can lead to increased nerve activity on both sides of the brain. However, the auditory cortex responds more strongly to stimulation of the contralateral ear (Langers et al., 2007; Smits et al., 2007). Thus, for RPT patients, it may be reasonable to find significantly increased CBF in the left PAC and SAC compared with HCs.

There are several limitations in this study. First, this study included a relatively small sample size, especially for LPT patients. PT accounts for only 4% of the tinnitus population, and there are more RPT patients than LPT patients (Wang et al., 2014; Zhang et al., 2019). Therefore, PT is a relatively rare entity. More patients will be included in future studies. Second, a single etiology of PT was included in this study. Sigmoid sinus wall dehiscence with or without diverticulum is the most common abnormality in PT in our daily work. In the future, PT patients with different etiologies should be included to investigate whether the etiology affects the results. Third, the change in gray matter volume may affect the measurement of CBF. Prior publications have confirmed that no difference in brain volume exists between PT patients and HCs (Han et al., 2014; Lv et al., 2016a,b, 2017, 2018). Thus, we did not explore structural changes in PT patients in this study. Finally, this is a cross-sectional study. We will explore the CBF changes and lateralization effects in PT patients after surgery success and further reveal the influence of PT on brain perfusion and lateralization effects.

## Conclusion

In conclusion, increased CBF in the left IPG is a common feature in both RPT and LPT patients. The lateralization effects of the auditory cortices may be a physiological characteristic of the normal brain regardless of the perceived side of tinnitus and the presence of tinnitus. Our findings not only increase our understanding of the neurological pathophysiology of PT but also provide a new method for future studies on PT.

## Data Availability Statement

The raw data supporting the conclusions of this article will be made available by the authors, without undue reservation.

## Ethics Statement

The studies involving human participants were reviewed and approved by Research Ethics Committee of Beijing Friendship Hospital, Capital Medical University. The patients/participants provided their written informed consent to participate in this study.

## Author Contributions

XL, PZ, HD, XQ, and HL performed the experiment and collected, analyzed, or interpreted the data involved in the study. XL preprocessed image data, performed the statistical results, and drafted the manuscript. PZ, HD, HL, and ZW designed the study and ensured the questions related to all aspects of the work. PZ, ZY, SG, and ZW gave critical comments on the manuscript. All authors contributed to the article and approved the submitted version.

## Conflict of Interest

The authors declare that the research was conducted in the absence of any commercial or financial relationships that could be construed as a potential conflict of interest.

## Footnotes

1 http://www.fil.ion.ucl.ac.uk/spm
